# Adherence to a healthy lifestyle and its association with cognitive impairment in community-dwelling older adults in Shanghai

**DOI:** 10.3389/fpubh.2023.1291458

**Published:** 2023-12-18

**Authors:** Yiqiong Qi, Ziwei Zhang, Xiya Fu, Peipei Han, Weixin Xu, Liou Cao, Qi Guo

**Affiliations:** ^1^Department of Rehabilitation Medicine, Shanghai University of Medicine and Health Sciences Affiliated Zhoupu Hospital, Shanghai, China; ^2^Department of Sport Rehabilitation, Shanghai University of Sport, Shanghai, China; ^3^Department of Rehabilitation Medicine, Shanghai University of Medicine and Health Sciences, Shanghai, China; ^4^Fujian Provincial Hospital, Fujian, China; ^5^Department of Laboratory Medicine of Central Hospital of Jiading District Shanghai Affiliated to Shanghai University of Medicine and Health Sciences, Shanghai, China; ^6^Department of Nephrology, Molecular Cell Lab for Kidney Disease, Ren Ji Hospital, Shanghai Jiao Tong University School of Medicine, Shanghai, China; ^7^Department of Rehabilitation Medicine, School of Health, Fujian Medical University, Fuzhou, China

**Keywords:** healthy lifestyle, cognitive impairment, physical activity, waist-to-hip ratio, community-dwelling older adults

## Abstract

**Introduction:**

There is a growing body of recent literature linking the association of specific or multiple lifestyles with cognitive impairment, but most of these studies have been conducted in Western populations, and it is necessary to study multiple lifestyles and cognitive abilities in different populations, with the primary population of this study being a select group of community-dwelling older adults in Shanghai, China.

**Methods:**

The sample included 2,390 community-dwelling Chinese participants. Their cognitive function was assessed using the Mini-Mental State Examination (MMSE). We defined a healthy lifestyle score on the basis of being non-smoking, performing ≥210 min/wk moderate/vigorous-intensity physical activity, having light to moderate alcohol consumption, eating vegetables and fruits daily, having a body mass index (BMI) of 18.5–23.9 kg/m^2^, and having a waist-to-hip ratio (WHR) <0.90 for men and <0.85 for women, for an overall score ranging from 0 to 6.

**Results:**

Compared with participants with ≤2 healthy lifestyle factors, the adjusted odds ratio (OR) and 95% confidence interval (CI) for participants with 4, 5, and 6 healthy lifestyle factors were 0.53 (95% CI, 0.29–0.98), 0.40 (95% CI, 0.21–0.75), and 0.36 (95% CI, 0.16–0.79), respectively. Only WHR (OR = 0.54, 95% CI = 0.37–0.78) and physical activity (OR = 0.69, 95% CI = 0.51–0.92) were associated with cognitive impairment. A healthy lifestyle correlated with overall cognition (β = 0.066, orientation (β = 0.049), language ability (β = 0.060), delayed recall (β = 0.045) and executive function (β = 0.044) (*P* all < 0.05).

**Conclusion:**

The study provides evidence on an inverse association between healthy lifestyles and cognitive impairment. We investigated whether healthy lifestyle was related to specific cognitive functions to provide a theoretical basis for accurate clinical prescription.

## 1 Introduction

Population aging has become a significant trend in China's social development, and the degree of population aging in China has further intensified. With increasing population aging due to improvements in life expectancy, there will be a large population of older adults with a high prevalence for cognitive impairment (1, 2). With an annual increase of more than 0.36 million, the total number of patients with cognitive impairment in China is expected to reach 48.68 million by 2060 (3). This condition has high prevalence, many risk factors, a complex etiology, and causes great harm to the older adults population (4). Thus, early screening, diagnosis, and intervention for cognitive impairment in older adults individuals are of great importance.

An emerging number of studies have linked modifiable lifestyles to the prevention and management of cognitive impairment. The majority of evidence has linked specific lifestyle factors with cognitive function, such as non-smoking (5), moderate alcohol consumption (6), physical activity (7), healthy diets (8), and low adiposity (9). However, lifestyle factors are not isolated; they tend to cluster, so potentially synergistic effects might occur (10). Each lifestyle factor is scored and summed up to construct a healthy lifestyle score; higher scores indicate a healthier lifestyle. A combined healthy lifestyle score is a strong indicator that plays a crucial role in factors regulating the onset of cognitive impairment. A previous study showed that adherence to a healthy lifestyle defined by a combination of these modifiable factors was related to up to roughly a 32% reduction in dementia incidence, in white populations from developed countries (11). Similarly, in two prospective cohort studies of older people, an increase in healthy lifestyles was found to be associated with a decrease in the risk of Alzheimer's disease (12). However, little is known whether such protective effects persist in different populations like the Asian population. Many health behaviors are based on culture and economy, and therefore, conclusions drawn from studies in western populations may not be generalizable to eastern populations. With economic and social development, Chinese people's lifestyles are constantly changing. In addition, the relatively low fixed mobility of older adults living in the Shanghai community lends itself to long-term observation. Therefore, the healthy lifestyles of older adults in the community deserve attention.

The authors' previous studies have shown that both physical performance (13, 14) and obesity (15) are associated with cognitive impairment. Hence, the purpose of this study was to examine the association between a healthy lifestyle composed of six modifiable lifestyle factors [body mass index (BMI), waist-to-hip ratio (WHR), physical activity, smoking status, alcohol consumption, and fruit and vegetable consumption] and the prevalence of cognitive impairment among community-dwelling older adults in Shanghai. Moreover, it also investigated whether having a healthy lifestyle was related to specific cognitive functions to provide a basis for clinical precision prescription. The goal of our study is to shed light on potential health intervention strategies to provide a theoretical basis for the early prevention and identification of cognitive impairment to establish an early warning model of cognitive impairment.

## 2 Materials and methods

### 2.1 Study subjects

Our research population included residents from Chongming and Hongkou District, Shanghai, China, who had joined China's national free physical examination program between March 2019 and September 2021. Individuals were included if they met the following criteria: (1) older adults ≥ 65 years old; (2) those who had lived in the community for at least 1 year; and (3) those who were willing to participate in this study. The exclusion criteria were as follows: (1) having Alzheimer's disease and other forms of dementia (*n* = 5); (2) having other cognitive impairments caused by neurodegenerative diseases, brain trauma, epilepsy, tumors, or infection (*n* = 2); (3) an inability to complete the exam due to vision or hearing impairments (*n* = 7) and (4) subjects with missing essential data (*n* = 35). The remaining 2,390 participants were included in the final analytic sample. All the participants had completed a questionnaire, had physical measurements taken, and had completed a written informed consent form.

### 2.2 Assessment of cognitive function

Cognitive function was evaluated by using the MMSE in this study, which was validated for Chinese seniors. It includes 30 items, and the score ranges from 0 to 30 points, with higher scores indicating better cognitive performance. The MMSE includes a broad set of cognitive domains that measure the following: orientation to time (five points), orientation to place (five points), registration (three points), attention and calculation (five points), recall (three points), and language (nine points) (16). Considering the significant correlation between education level and MMSE scores, in China, the cut-off points for defining cognitive impairment are 17/18, 20/21, and 24/25 for illiterate people, people with primary education, and people with education above middle school, respectively (17).

### 2.3 Assessment of lifestyle factors

The participants reported a range of lifestyle factors in the baseline questionnaire. Questions about tobacco smoking included the frequency, type, and amount of tobacco smoked per day for current smokers and years since quitting for former smokers. Regarding alcohol consumption, the questions included the frequency of alcohol consumption and the number of different types of alcohol consumed in the last month. The conversion method for the alcohol content in various wines is as follows: alcoholic beverages (ml)^*^alcohol content (%)^*^0.8. Physical activity was assessed using the short form of the International Physical Activity Questionnaire (IPAQ), and we described the methods of the IPAQ in detail in a previous study (18). Lastly, the total number of minutes of moderate- and high-intensity physical activity per week was calculated. Questions about diet included “Do you eat 2 or more servings of fruit and vegetables every day?”, and participants need to answer yes or no. Professionally trained staff use calibrated instruments to measure weight, height, waist circumference, and hip circumference. BMI was calculated by dividing the weight in kilograms by the height in square meters. WHR is the ratio of the waist circumference to the hip circumference.

### 2.4 Assessment of covariates

The interviewees collected participant sociodemographic variables, including age, sex, educational level, marital status, living status, and self-reported medical conditions, through a paperless questionnaire survey. According to the participants' responses to their medical histories, their past doctors' diagnoses, current or historical drug treatment programs, and history of physical diseases, including type 2 diabetes mellitus (T2DM), hypertension, hyperlipidaemia, stroke, and heart disease, was evaluated. Participants who reported having a history of more than one fall during the past year were categorized as “fallers” (19).

### 2.5 Classification of healthy lifestyle categories

Based on the evidence (20–23), guidelines (24), and expert knowledge, to address the health benefits of lifestyle factors in preventing cognitive impairment, we considered six healthy lifestyle behaviors a priori: (1) For smoking, the healthy group was people who do not smoke at present; (2) for alcohol consumption, the healthy group was those who drank less than once weekly, weekly but not daily drinkers, or daily drinkers with an intake of < 30 g of pure alcohol in men or < 15 g in women; (3) for physical activity, we classified the healthy group as participating in at least 30 min of moderate or vigorous activity daily (3.5 h/week); (4) for dietary habits, eating both fruit and vegetables daily was considered to be healthy; (5) for general adiposity measured by BMI, the healthy group was defined as those who had a BMI of 18.5–3.9 kg/m^2^, the standard classification of normal weight specific to Chinese people; and (6) for central adiposity measured by WHR, the healthy group was defined as those who had a WHR < 0.90 in men and < 0.85 in women.

For each healthy lifestyle factor, the participants received a score of 1 if they met the criteria for health and 0 if they did not meet the criteria. The sum of these six scores yielded a final score within the range from 0 to 6, with higher scores indicating a healthier lifestyle. Because of the small number of participants scoring 0 (*n* = 1) and 1 (*n* = 8), we categorized the healthy lifestyle scores into five groups: 0–2, 3, 4, 5. and 6.

### 2.6 Statistical analysis

Continuous variables were expressed as the means ± standard deviation, and classified variables were expressed as a percentage (%). Analysis of variance and Kruskal-Wallis test corrected by Bonferroni were used for continuous variables and χ^2^ test was used for categorical variables. Logistic regression models were used to examine the relationship between a healthy lifestyle and cognitive impairment. Linear regression models were used to analyse the relationship between a healthy lifestyle, overall cognition, and various fields. The adjusted variables included participant age, sex, Illiteracy, job type, diabetes, hypertension, hyperlipidaemia, and faller status. All the statistical analyses were performed using SPSS version 26.0, and a *P*-value of < 0.05 was considered statistically significant.

## 3 Results

**Figure 1:**
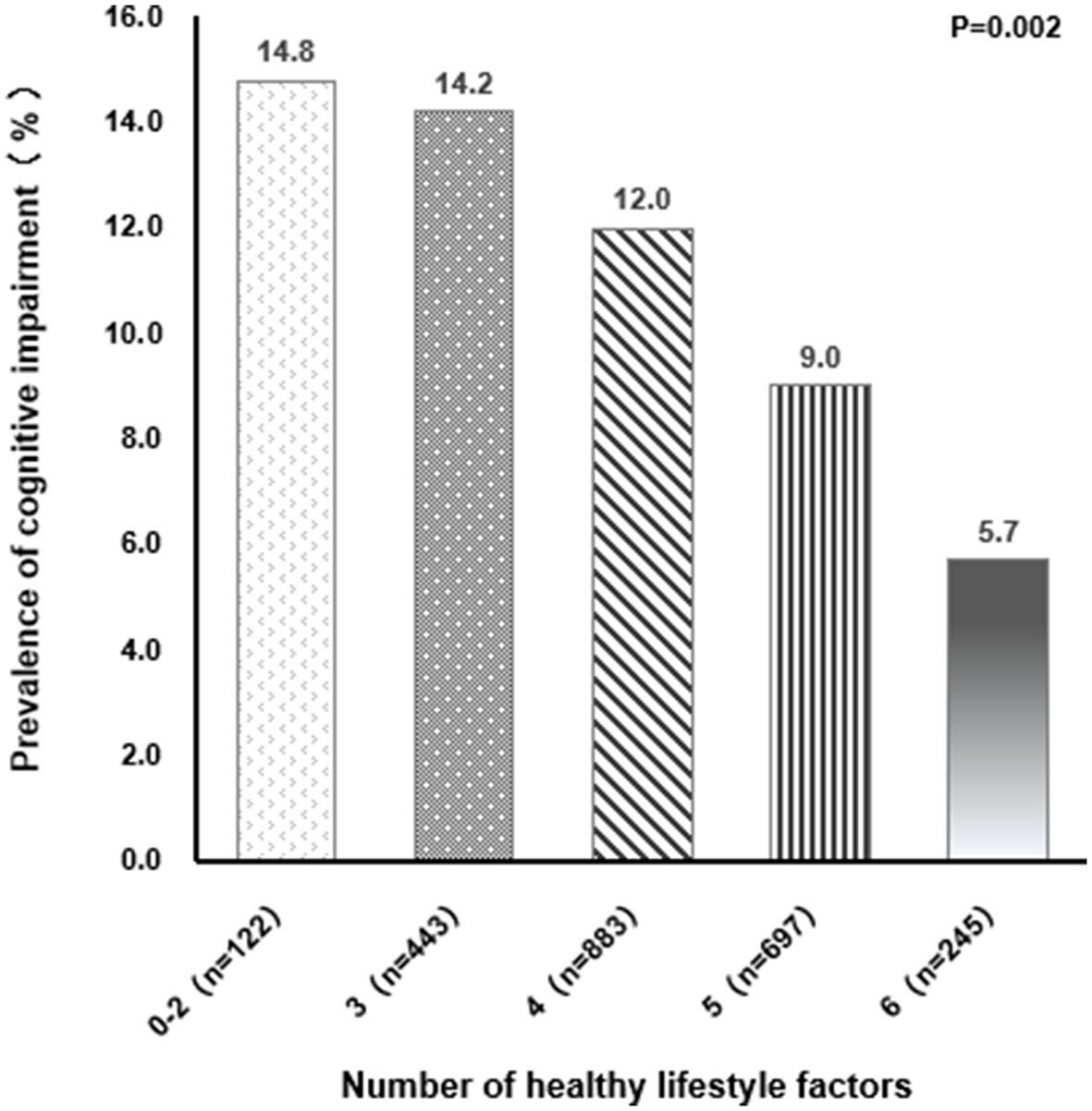
Relationship between different healthy lifestyle factors and prevalence of cognitive impairment.

Table 1 shows the participants' characteristics as stratified by healthy lifestyle factors. The numbers and proportions of all participants with healthy lifestyle scores, from lowest to highest, were 122 (5.10%), 443 (18.54%), 883 (36.95%), 697 (29.16%), and 245 (10.25%). Participants who were women, younger, more educated, and mental workers were more likely to adhere to a healthy lifestyle. With regard to healthy lifestyle factors, the majority of older adults (95.65%) consume fruits and vegetables daily, 88.62% were normal drinkers, 87.03% were non-smokers, 69.41%were engaging in physical activity regularly, 49.33% were normal weight and 30.54% were well-proportioned body. Compared with those with 0–2 healthy lifestyle factors, those with six healthy lifestyle factors were less likely to suffer from diabetes, hypertension, and hyperlipidaemia. Participants with more healthy lifestyle factors had a lower prevalence of cognitive impairment (*P* = 0.002, Figure 1). Table 2 shows the results of the logistic regression analysis investigating the association between a healthy lifestyle and cognitive impairment. The prevalence of cognitive impairment decreased along with an increasing number of healthy lifestyle factors. Compared with participants with ≤ 2 healthy lifestyle factors, the adjusted odds ratio (OR) and 95% confidence interval (CI) for participants with 4, 5, and 6 healthy lifestyle factors were 0.53 (95% CI, 0.29–0.98), 0.40 (95% CI, 0.21–0.75), and 0.36 (95% CI, 0.16–0.79), respectively.

**Table 1:** Baseline characteristics of 2,390 participants according to the healthy lifestyle score.

**Characteristics**	**Healthy lifestyle score (points)**					***p*-value**
	**0–2 (*****n*** = **122)**	**3 (*****n*** = **443)**	**4 (*****n*** = **883)**	**5 (*****n*** = **697)**	**6 (*****n*** = **245)**	
Age (y)	71.81 ± 6.05	72.66 ± 6.18	72.15 ± 5.98	72.03 ± 5.97	71.15 ± 5.33^bcd^	0.032
Male (%)	106 (86.9)	269 (60.7)^a^	307 (34.8)^ab^	246 (35.3)^ab^	92 (37.6)^ab^	< 0.001
BMI (kg/m^2^)	25.93 ± 2.92	25.51 ± 3.44	24.66 ± 3.40^ab^	21.88 ± 2.30^abc^	21.28 ± 1.55^abcd^	< 0.001
Education level (%)						< 0.001
Illiteracy	4 (3.4)	46 (10.3)	92 (10.5)	55 (7.9)	11 (4.4)	
Primary school	76 (63.9)	261 (58.5)	569 (65.1)	430 (61.9)	138 (55.4)	
Above middle school	39 (32.8)	139 (31.2)	213 (24.4)	210 (30.2)	100 (40.2)	
Monthly income (%)						0.060
< 1,000 RMB	6 (5.0)	20 (4.5)	44 (5.0)	31 (4.4)	7 (2.9)	
1,000–3,000 RMB	47 (38.8)	176 (39.9)	361 (41.1)	264 (37.9)	75 (30.6)	
3,000–5,000 RMB	17 (14.0)	64 (14.5)	137 (15.6)	110 (15.8)	31 (12.7)	
>5,000 RMB	51 (42.1)	181 (41.0)	337 (38.3)	292 (41.9)	132 (53.9)	
Job type (%)						0.001
Mental labor	25 (20.5)	116 (26.2)	195 (22.3)	176 (25.5)	85 (34.8)	
Manual labor	74 (60.7)	271 (61.3)	559 (64.0)	416 (60.2)	116 (47.5)	
Both	23 (18.9)	55 (12.4)	119 (13.6)	99 (14.3)	43 (17.6)	
Widowed (%)	16 (13.1)	70 (15.8)	170 (19.3)	119 (17.1)	41 (16.7)	0.333
Living alone (%)	20 (16.4)	67 (15.1)	139 (15.7)	95 (13.6)	37 (15.1)	0.809
Faller (%)	63 (51.6)	240 (54.2)	497 (56.3)	349 (50.1)^c^	103 (42.0)^bcd^	0.001
Diabetes (%)	34 (27.9)	115 (26.0)	202 (22.9)	135 (19.4)^ab^	21 (8.6)^abcd^	< 0.001
Hypertension (%)	93 (76.2)	335 (75.6)	638 (72.3)	434 (62.3)^abc^	118 (48.2)^abcd^	< 0.001
Hyperlipidemia (%)	54 (44.3)	196 (44.2)	390 (44.2)	261 (37.4)^bc^	89 (36.3)^bc^	0.019
Heart disease (%)	34 (27.9)	138 (31.2)	280 (31.7)	188 (27.0)	66 (26.9)	0.221
Stroke (%)	26 (21.3)	93 (21.0)	198 (22.4)	130 (18.7)	35 (14.3)	0.053
Cognitive impairment (%)	18 (14.8)	63 (14.2)	106 (12.0)	63 (9.0)^b^	14 (5.7)^abc^	0.002
Having healthy lifestyle factors (%)						< 0.001
Former or never smoke	39 (32.0)	326 (73.6)^a^	809 (91.6)^ab^	661 (94.8)^abc^	245 (100.0)^abcd^	
Moderate or never drink	54 (44.3)	338 (76.3)^a^	814 (92.2)^ab^	667 (95.7)^abc^	245 (100.0)^abcd^	
Physical activity ≥ 3.5 h/week (moderate or vigorous)	37 (30.3)	169 (38.1)	641 (72.6)^ab^	567 (81.3)^abc^	245 (100.0)^abcd^	
BMI within 18.5–23.9 kg/m^2^	8 (6.6)	63 (14.2)^a^	284 (32.2)^ab^	579 (83.1)^abc^	245 (100.0)^abcd^	
Daily vegetables and fruits	98 (80.3)	403 (91.0)^a^	853 (96.6)^ab^	687 (98.6)^abc^	245 (100.0)^abc^	
WHR < 0.9 in men, < 0.85 in women	0 (0.0)	30 (6.8)^a^	131 (14.8)^ab^	324 (46.5)^abc^	245 (100.0)^abcd^	

BMI, body mass index; WHR, waist-to-hip ratio; Data are presented as mean ± SD or *n* (%).

^a^Compared with score = 0–2 groups, *p* < 0.05.

^b^Compared with score = three groups, *p* < 0.05.

^c^Compared with score = four groups, *p* < 0.05.

^d^Compared with score = five groups, *p* < 0.05.

**Table 2:** Adjusted odds ratios for the association of healthy lifestyle score with cognitive impairment.

**Healthy lifestyle score**	**Unadjusted**		**Adjusted**	
	**OR (95% CI)**	***P***	**OR (95% CI)**	***P***
0–2	Ref.	–	Ref.	–
3	0.96 (0.54–1.69)	0.882	0.67 (0.36–1.26)	0.213
4	0.79 (0.46–1.35)	0.388	0.53 (0.29–0.98)	0.042
5	0.57 (0.33–1.01)	0.054	0.40 (0.21–0.75)	0.004
6	0.35 (0.17–0.73)	0.005	0.36 (0.16–0.79)	0.012

Adjusted by age, sex, education level, job type, diabetes, hypertension, hyperlipidemia, faller.

OR, Odds ratios; CI, confidence interval.

Associations between individual healthy lifestyle factors and cognitive impairment are shown in Table 3. Only WHR (OR = 0.54, 95% CI = 0.37–0.78) and physical activity (OR = 0.69, 95% CI = 0.51–0.92) were significantly associated with cognitive impairment. Furthermore, the relationship between a healthy lifestyle, overall cognition, and various fields was analyzed. The healthy lifestyle score correlated with the total score of the MMSE, orientation, language ability, delayed recall and executive function (Table 4).

**Table 3:** Logistic regression model for association of healthy lifestyle factors and cognitive impairment.

**Lifestyle factors**	**Unadjusted**		**Adjusted**	
	**OR (95% CI)**	***P***	**OR (95% CI)**	***P***
**Body mass index**				
< 18.5 or ≥ 23.9 kg/m^2^ (*n* = 1,211)	Ref.	–	Ref.	–
18.5–23.9 kg/m^2^ (*n* = 1,179)	0.95 (0.73–1.22)	0.673	1.00 (0.75–1.32)	0.973
**WHR**				
≥0.9 (men), ≥0.85 (women) (*n* = 1660)	Ref.	–	Ref.	–
< 0.9 (men), < 0.85 (women) (*n* = 730)	0.43 (0.31–0.60)	< 0.001	0.54 (0.37–0.78)	0.001
**Vegetables and fruits**				
Less than daily (*n* = 104)	Ref.	–	Ref.	–
Daily (*n* = 2,286)	0.58 (0.34–0.97)	0.040	0.57 (0.32–1.02)	0.057
**Smoking**				
Current (*n* = 310)	Ref.	–	Ref.	–
Former or never (*n* = 2,080)	1.23 (0.82–1.85)	0.309	0.71 (0.44–1.16)	0.174
**Alcohol consumption**				
Not moderate (*n* = 272)	Ref.	–	Ref.	–
Moderate or never (*n* = 2,118)	0.96 (0.65–1.43)	0.844	0.99 (0.62–1.57)	0.958
**Physical activity**				
< 3.5 h/week (moderate or vigorous) (*n* = 731)	Ref.	–	Ref.	–
≥3.5 h/week (moderate or vigorous) (*n* = 1,659)	0.66 (0.50–0.85)	0.002	0.69 (0.51–0.92)	0.013

Adjusted by age, sex, education level, job type, diabetes, hypertension, hyperlipidemia, faller.

HR, hazard ratio; CI, confidence interval.

**Table 4:** Linear regression analysis of healthy lifestyle scores and cognitive domain scores.

**Variables**	**Unadjusted analysis**				**Adjusted analysis**			
	***B***	**95%CI**	**SE**	***P*** **-value**	***B***	**95%CI**	**SE**	***P*** **-value**
MMSE score	0.323	0.160 to 0.487	0.079	< 0.001	0.268	0.129 to 0.408	0.071	< 0.001
**MMSE subscores**								
Orientation	0.083	0.026 to 0.140	0.029	0.004	0.069	0.019 to 0.119	0.026	0.007
Short-term memory	0.027	0.007 to 0.046	0.010	0.008	0.018	−0.002 to 0.038	0.010	0.084
Attention and calculation	0.059	−0.002 to 0.119	0.031	0.057	0.053	−0.005 to 0.111	0.030	0.076
Delayed recall	0.061	0.015 to 0.106	0.023	0.009	0.051	0.004 to 0.097	0.024	0.034
Language ability	0.070	0.032 to 0.108	0.019	< 0.001	0.057	0.023 to 0.090	0.017	0.001
Executive function	0.021	0.004 to 0.038	0.009	0.014	0.018	0.002 to 0.035	0.009	0.030

Adjusted by age, sex, education level, job type, diabetes, hypertension, hyperlipidemia, faller.

CI, confidence interval; SE, standard error.

## 4 Discussion

In this cross-sectional survey of older adults in the community, a significant association was observed between a healthy lifestyle score defined by six lifestyle factors and cognitive impairment. This linear association, which exists independently of gender and age, also suggests that participants with a greater number of healthy lifestyle factors had a gradually decreased (67.1%–35.7%) prevalence of adverse outcomes. Moreover, we found that appropriate WHR and regular physical activity were associated with cognitive impairment. Specifically, the relationships between a healthy lifestyle and specific cognitive functions were analyzed. The healthy lifestyle score was found to correlate with the total score for MMSE, orientation, language ability, delayed recall and executive function. To the best of our knowledge, this is the first study to explore the relationship between healthy lifestyle and specific cognitive functions among community-dwelling older adults in Shanghai.

The inverse association between healthy lifestyle and cognitive function observed in our study has been previously reported in other countries (25, 26). One example comes from a Korean cross-sectional study that used a similar definition of a healthy lifestyle and showed that participants with three or more healthy lifestyle factors (i.e., non-smoking, normal drinking, physical activity, and normal weight) were at lower prevalence for cognitive impairment than participants with zero healthy lifestyle factors (26). Similarly, a cohort study from the United Kingdom showed a reduced prevalence of cognitive impairment among men with four to five healthy lifestyle factors (i.e., non-smoking, an acceptable BMI, a high fruit and vegetable intake, regular physical activity, and low/moderate alcohol intake) (25). Our study extends these previous studies by showing the association between a healthy lifestyle and cognitive impairment among community-dwelling older adults in Shanghai, where a healthy lifestyle may be different from that of older adults in other countries and the disease burden associated with cognitive impairment is increasing. The healthy lifestyle scores constructed in previous studies involved different numbers of lifestyle factors, but BMI, physical activity, smoking, and alcohol consumption were frequently used (5–9). Despite the heterogeneity in the components of the healthy lifestyle score, the present study and previous studies provide robust evidence for the association between healthy lifestyle and cognitive impairment.

The impact of a healthy lifestyle may vary due to the interaction of many factors in different countries. Firstly, the dietary habits in some European countries tend to favor foods that are high in fat, sugar, and salt. This could potentially contribute to a significant increase in health issues such as obesity (27). A recent study on Europeans aged 65 and above revealed an obesity rate of 20.9% (28). In this study, the obesity rate among the older adults is 10.54%, which is comparable to another study conducted on the older adults population in China with an obesity rate of 11.53% (29). This may be attributed to healthier dietary habits, as the majority of participants (95.65%) in this study consume fruits and vegetables on a daily basis. Secondly, the current smoking prevalence in this study is 12.97%, which is significantly lower than the average smoking rate in ten European countries (20.2%) (30). This could be attributed to a higher proportion of females in this study, with a smoking rate of 29.71% for males and 0.51% for females. This indicates that a greater awareness of the importance of smoking cessation among the older adults, possibly influenced by health education through media, hospitals, communities, and increased self-awareness of health among the older adults. Thirdly, 69.41% of the participants in this study were engaging in physical activity regularly, which may be attributed to their residence in suburban areas, where the majority (60.08%) were involved in physically demanding occupations. This aligns with our previous research, where the proportion of farmers was as high as 53.45% (13). In contrast, many studies focusing on Western populations tend to emphasize leisure-time physical activities, often of moderate intensity (31). Overall, participants recruited from some communities in Shanghai had a more active lifestyle, which differed from participants in other areas.

Moreover, we found that appropriate WHR and regular physical activity were associated with cognitive impairment better than other lifestyle factors. This finding is similar to previous studies. A large Korean study found that men with a waist circumference of 90 cm or greater and women with a waist circumference of 85 cm or greater are considered to be at a higher probability for developing dementia (32). In a large older adults population study, the researchers found that higher WC and WHR were significantly associated with a higher prevalence of cognitive impairment (33). There are several potential mechanisms through which WHR may influence cognitive impairment (34). These include neurodegenerative processes, vascular factors, and metabolic processes that impact brain structures. For example, high WHR has been associated with increased blood pressure, dyslipidemia, and insulin resistance, all of which negatively impact vascular health. Impaired vascular function can result in reduced blood flow to the brain, leading to decreased oxygen and nutrient supply. This compromised blood flow can contribute to the development of cognitive impairment (35). In summary, the relationship between WHR and cognitive decline is likely complex and multifactorial, involving interactions between genetic, environmental, and lifestyle factors. Similarly, strong observational data have identified physical activity as a potent lifestyle factor that plays a critical role in alleviating age-related cognitive decline across the lifespan (36, 37). A previous SAGE cohort study by Huang et al. investigated the contemporaneous association of five modifiable lifestyle factors with age-related cognitive decline and found that higher levels of physical activity were positively associated with all cognitive domains (38). A recent study among women aged 65 and over showed that an extra 31 min of moderate to strenuous physical activity every day reduced the prevalence of mild cognitive impairment or dementia by 21% (39). In addition, a previous study by our team showed that physical performance (grip strength, TUGT, and 4-m walking speed) correlated with MCI (13). It is hypothesized that neural and vascular adaptations to physical activity improve cognition by promoting neurogenesis, angiogenesis, and synaptic plasticity, decreasing proinflammatory processes and reducing cellular damage due to oxidative stress (40). We did not find evidence for significant associations between individual factors other than WHR and physical activity and cognitive impairment, but this does not mean that we do not need to account for these factors, because the more healthier lifestyle factors there were, the lower the prevalence of cognitive impairment. Consistent with the results of this study, there are also studies that found no significant association between diet, alcohol consumption, and smoking with cognition (41–43). The diversity in populations and contextual factors highlights the complexity of these associations. Previous studies have also considered the combined effect of multiple health-related lifestyles, not all of which were significantly associated with the outcome (38, 44). The mechanisms that underlie the relationship between lifestyle factors and cognition are not yet fully understood. Focusing on multiple integrated lifestyles and the risk of developing cognitive impairment is more predictive than focusing on a single lifestyle, and the findings are easily understood by the public and translated into practical health-improving behaviors. In future studies, other measures could be explored, including consideration of different weightings for different lifestyle domains, or participants' subjective perceptions of their combined lifestyle health.

Further analysis of the relationship between healthy lifestyle scores and overall cognition and various cognitive domains revealed that healthy lifestyle correlated with overall cognition, orientation, language, delayed recall and executive function but did not correlate with short-term memory, attention, calculation. We speculate that perhaps short-term memory can be improved by acquired training and has little association with lifestyle. A population-based study of participants ≥ 65 years old from the Hellenic Longitudinal Investigation of Ageing and Diet (HELIAD) showed that each lifestyle factor was differentially associated with domain-specific cognitive performance (45). Huang et al. found that higher vegetable and fruit consumption as well as higher levels of physical activity were positively associated with all cognitive domains (38). A study exploring the relationship between lifestyle factors and neurocognitive function in older adults showed that greater aerobic capacity and daily physical activity were associated with better executive functioning (46). However, others have shown that aerobic fitness improvements are associated with small volume increases within mesial temporal brain structures that are preferentially important for memory but not executive function (47). The discrepancies in the literature may be due in part to differences in the characteristics of the study populations and in the measures used to assess neurocognition. The mechanism may be that exercise increases cerebral blood perfusion, elevates peripheral brain-derived neurotrophic factor and insulin-like growth factor 1 levels, stimulates the secretion of catecholamine neurotransmitters in the brain, and promotes the integrity of brain structures, especially the gray matter in the prefrontal and medial temporal lobe regions (48). It is necessary for future research to focus on the specific dimensions of cognition to provide the basis for accurate clinical prescription.

The current study had several strengths. We comprehensively investigated the relationship between healthy lifestyle and cognitive impairment and examined whether and which healthy lifestyle factors drive this relationship. This study was the first to examine the relationship between healthy lifestyle and specific cognitive functions, and the applicability of this multidimensional lifestyle evaluation system among community-dwelling older adults in Shanghai was also further tested. Several limitations of the current study should also be considered. First, this study is based on a cross-sectional design. Thus, it is not possible to elucidate clear causal associations between healthy lifestyle and cognitive impairment in older adults. In the future, it would be valuable to perform a prospective study to provide more robust evidence regarding causality. Equally important, we will incorporate more indicators to establish a more comprehensive evaluation system for lifestyle. We will also conduct healthy lifestyle intervention experiments in the older adults population in Shanghai when possible. Second, this study was limited by its reliance on self-reported lifestyle factors; measurement errors are inevitable. Third, all participants in this study were relatively healthy, and this selection may constitute selective survival and healthy selection bias. Furthermore, MMSE is a screening tool and not very detailed and sensible when separated by cognitive area, consideration should be given to adding specialized scales to rate different domains of cognition, such as the MoCA, CDT, ADAS-Cog.

Our findings suggest that adhering to a greater number of healthy lifestyle factors may protect against cognitive decline. Appropriate WHR and regular physical activity are protective against cognitive decline in older adults. We also investigated whether healthy lifestyle was related to specific cognitive functions to provide a theoretical basis for accurate clinical prescription. In the context of aging and with the growing number of patients with cognitive impairment, even modest positive effects on cognitive function related to greater adherence to an overall healthy lifestyle could yield significant public health benefits if they can be cost-effectively delivered on a large scale. Therefore, these factors must be promoted in the older adults as a strategy to delay or prevent cognitive impairment.

## Data availability statement

The original contributions presented in the study are included in the article/supplementary material, further inquiries can be directed to the corresponding author.

## Ethics statement

The studies involving humans were approved by the Ethics Committee at the Shanghai University of Medicine and Health Sciences (2019-WJWXM-04-310108196508064467). The studies were conducted in accordance with the local legislation and institutional requirements. The participants provided their written informed consent to participate in this study.

## Author contributions

YQ: Writing—original draft, Writing—review & editing, Data curation, Investigation. ZZ: Writing—original draft, Writing—review & editing, Investigation. XF: Writing—original draft, Writing—review & editing, Investigation. PH: Conceptualization, Writing—review & editing. WX: Writing—review & editing. LC: Writing—review & editing. QG: Methodology, Supervision, Conceptualization, Writing—review & editing.
